# Neoadjuvant Anlotinib and chemotherapy followed by minimally invasive esophagectomy in patients with locally advanced esophageal squamous cell carcinoma: Short-term results of an open-label, randomized, phase II trial

**DOI:** 10.3389/fonc.2022.908841

**Published:** 2022-08-02

**Authors:** Ying-Jian Wang, Kun-Kun Li, Xian-Feng Xie, Tao Bao, Zhi-Peng Hao, Jiang Long, Shuai Wang, Zhao-Yang Zhong, Wei Guo

**Affiliations:** ^1^ Department of Thoracic Surgery, Daping Hospital, Army Medical University (Third Military Medical University), Chongqing, China; ^2^ Department of Cancer Center, Daping Hospital, Army Medical University (Third Military Medical University), Chongqing, China

**Keywords:** Anlotinib, chemotherapy, chemoradiotherapy, esophageal squamous cell carcinoma, minimally invasive esophagectomy, neoadjuvant therapy

## Abstract

**Background:**

Clinical benefits of neoadjuvant Anlotinib for locally advanced esophageal squamous cell carcinoma (ESCC) remains unclear. This study evaluated the efficacy and safety of neoadjuvant Anlotinib plus chemotherapy followed by minimally invasive esophagectomy (MIE) for the treatment of patients with locally advanced ESCC.

**Methods:**

Patients with locally advanced ESCC were randomly assigned to neoadjuvant Anlotinib combined with chemotherapy (Anlotinib group) or neoadjuvant chemoradiotherapy alone (nCRT group) with an allocation ratio of 1:1. The primary endpoint was the R0 surgical resection rate. Secondary endpoints included postoperative pathologic stage, complete response (CR) rate, and safety. Safety was assessed by adverse events (AEs) and postoperative complications.

**Results:**

From August 2019 to August 2021, 93 patients were assigned to the nCRT or Anlotinib group. Of the 93 patients, 79 underwent MIE and were finally included in the per-protocol set (nCRT group: n=39; Anlotinib group: n=40). The R0 resection rate was 97.4% for nCRT versus 100.0% for Anlotinib group (*p*>0.05). Compared with the nCRT group, patients in the Anlotinib group had shorter total operation duration (262.2 ± 39.0 vs. 200.7 ± 25.5 min, *p*=0.010) and less blood loss (161.3 ± 126.7 vs. 52.4 ± 39.3 mL, *p*<0.001). No significant differences were found in the postoperative pathologic stage between the Anlotinib group and nCRT group (all *p*>0.05). Besides, the incidences of AEs (80.0% vs. 92.3%) and postoperative complications (22.5% vs. 30.8%) were similar between the two groups (all *p*>0.05).

**Conclusions:**

Neoadjuvant Anlotinib plus chemotherapy had a similar safety profile and pathologic response, but better surgical outcomes than nCRT for locally advanced ESCC.

## Introduction

Esophageal cancer is the sixth most common cause of cancer-related deaths worldwide (1). Esophageal squamous cell carcinoma (ESCC) is the main subtype of esophageal cancer, which accounts for approximately 90% of the 456,000 incidents of esophageal cancer cases each year (2). For patients with locally advanced ESCC, esophagectomy is still the mainstay of current therapy. However, as a technically challenging procedure, esophagectomy alone is often accompanied by high rates of metastasis and recurrence (3). In this regard, multimodality regimens have been developed to improve survival.

Recently, increasing evidence has proved that patients can obtain a survival benefit from neoadjuvant therapy followed by surgery compared with surgery alone (4–7). The CROSS study demonstrated that patients who received preoperative neoadjuvant chemoradiotherapy (nCRT) had a better R0 resection rate, lower node-positive rate, and longer overall survival (OS) compared with surgery alone (4). The results from the JCOG9907 study indicated an improved 5-year OS rate of preoperative neoadjuvant chemotherapy (nCT) compared with surgery alone (55% vs. 43%, *p*= 0.04) (7). Based on the CROSS study and JCOG9907 study, preoperative nCRT and nCT have been adopted as standard treatments for patients with locally advanced ESCC in western and Asia countries, respectively (6). However, nCRT is significantly associated with an increased risk of postoperative morbidity and perioperative mortality, which may limit the clinical application of nCRT (8). Although many studies have confirmed that nCT is safe, and still has considerable room for improvement in pathological complete remission (pCR) of esophageal cancer (9, 10). Therefore, exploring new neoadjuvant regimens with manageable tolerability is crucial for patients with locally advanced ESCC to obtain more clinical benefits.

Anlotinib, a novel multitarget tyrosine kinase inhibitor (TKI), suppresses tumor angiogenesis and growth by targeting vascular endothelial growth factor receptor (VEGFR), fibroblast growth factor receptor (FGFR), platelet-derived growth factor receptor (PDGFR), and c-Kit (11). The antitumor activity of Anlotinib has been proved in several tumors (12–15). The 2021 European Society for Medical Oncology (ESMO) Congress showed that the addition of Anlotinib to neoadjuvant chemotherapy showed promising antitumor activity and manageable toxicity for patients with high-risk, early-stage triple-negative breast cancer (16). Besides, the phase II study of neoadjuvant Anlotinib yielded an objective response rate (ORR) of 76.9% in patients with locally advanced thyroid cancer (17). A preclinical study has demonstrated that combination therapy with Anlotinib and chemoradiotherapy exhibits the strongest antitumor response in patient-derived xenografts (PDXs) mouse models of ESCC compared with other treatment groups (radiotherapy only, chemoradiotherapy, and Anlotinib combined with radiotherapy) (18). A randomized phase II trial demonstrated that Anlotinib significantly prolonged progression-free survival (PFS) in patients with chemotherapy-refractory metastatic ESCC compared to placebo, with a manageable safety profile (19). However, evidence of Anlotinib as neoadjuvant therapy for locally advanced ESCC is still scanty currently.

There is no doubt that the high postoperative morbidity and mortality of esophagectomy results partly from the huge trauma caused by open surgery (20). In past decades, minimally invasive esophagectomy (MIE) tends to take the place of traditional open esophagectomy due to the advantage of less trauma and complications, as well as equal curative effect (21). Although several studies have assessed the efficacy of nCRT or nCT followed by MIE for patients with locally advanced ESCC, the long-term survival superiority of MIE has not been definitively established owing to the high demand for surgical skills (20, 22, 23). Here, we present a novel neoadjuvant regime of Anlotinib plus nCT by comparing it with nCRT alone, which is expected to reduce the technical requirement of the MIE procedure without offsetting the survival advantage. To the best of our knowledge, this is the first study evaluating the efficacy and safety of neoadjuvant Anlotinib plus chemotherapy followed by MIE for the treatment of patients with locally advanced ESCC.

## Materials and methods

### Study design

This study was a single-center, open-label, randomized, phase II trial (ChiCTR2000031228) to investigate the feasibility and safety of neoadjuvant Anlotinib and nCT followed by MIE in patients with locally advanced ESCC. Patients with locally advanced ESCC were recruited from August 2019 to August 2021. This study was conducted in conformance with Good Clinical Practice guidelines and the Declaration of Helsinki, and approved by the ethics committee of the Daping Hospital, Army Medical University (Third Military Medical University) (number: 202044). Written informed consents were provided to all patients.

### Patient eligibility

Previously untreated patients aged 18-75 years with histologically confirmed locally advanced ESCC (staging T2-T3, any N), had Eastern Cooperative Oncology Group (ECOG) performance status of 0-1, without distant metastasis, and did not participate in other clinical trials were eligible for this trial. Patients were ineligible if they had any significant medical condition which was thought unlikely to tolerate the neoadjuvant treatment, such as active tuberculosis, hepatic disease, immunodeficiency disease, another malignant tumor, as well as clinically-significant bone marrow, liver, renal function disorder. Patients who had hypersensitivity for paclitaxel or cisplatin, had a history of gastrointestinal perforation, fistula, or thrombus within 6 months before treatment, had mental disorders or history of psychotropic drug abuse, or had a major operation within 4 weeks before treatment or expected to require major operation during the treatment were also ineligible. Patients with long-term unhealed wounds or fractures, significant malnutrition, as well as pregnant or lactating patients at screening were also excluded from this trial.

### Procedures

**Figure 1:**
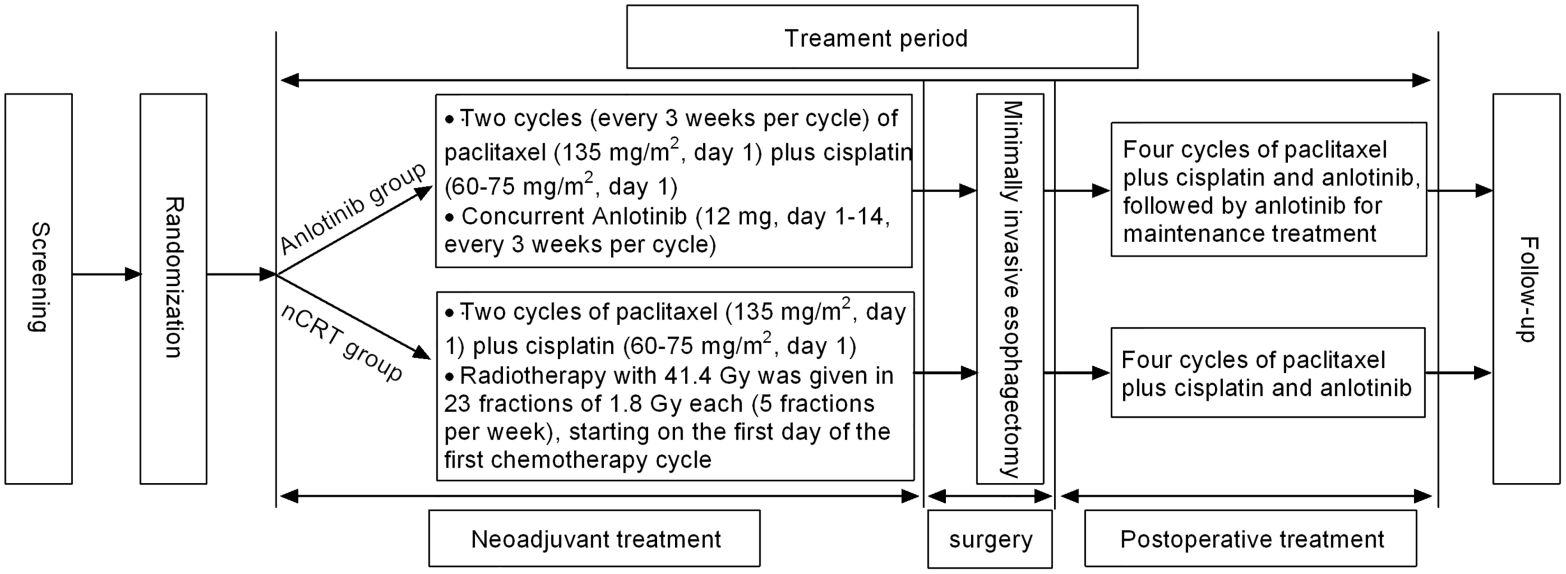
Study design. Anlotinib group, neoadjuvant Anlotinib combined with chemotherapy; nCRT group, neoadjuvant chemoradiotherapy.

Eligible patients were randomly (allocation ratio 1:1) assigned to receive neoadjuvant Anlotinib combined with nCT (Anlotinib group) or nCRT alone (nCRT group) *via* a computer-generated coding system (**Figure 1**).

In the Anlotinib group, preoperative neoadjuvant treatment consisted of 2 cycles (every 3 weeks per cycle) of paclitaxel (135 mg/m^2^, day 1) plus cisplatin (60-75 mg/m^2^, day 1) intravenously, and concurrent orally Anlotinib (12 mg, day 1-14, every 3 weeks per cycle; Chia-tai Tianqing Pharmaceutical Co., Ltd., Nanjing, China). In the nCRT group, patients received 2 cycles (every 3 weeks per cycle) of paclitaxel (135 mg/m^2^, day 1) plus cisplatin (60-75 mg/m^2^, day 1) intravenously, and concurrent radiotherapy for neoadjuvant treatment. Radiotherapy with 41.4 Gy was given in 23 fractions of 1.8 Gy each (5 fractions per week), starting on the first day of the first chemotherapy cycle. Dose adjustment of Anlotinib from 12 mg to 10 mg or 8 mg would be performed once grade 3-4 adverse events (AEs) occurred. Besides, suspension and discontinuation of Anlotinib were allowed when patients experienced disease progression or unacceptable toxicity.

MIE was scheduled 4-6 weeks after the completion of neoadjuvant Anlotinib combined with nCT, or nCRT alone. Preoperative restaging comprised of physical examination, standard laboratory tests, esophagogastroduodenoscopy with endoscopic ultrasound (EUS), pulmonary function tests, and neck-thorax-abdomen computed tomography (CT). Patients in the Anlotinib group postoperatively received 4 cycles of paclitaxel plus cisplatin and Anlotinib, followed by Anlotinib for maintenance treatment. In the nCRT group, postoperative therapy only consisted of 4 cycles of paclitaxel plus cisplatin.

### Endpoints

The primary endpoint was the R0 surgical resection rate. The R0 resection was defined as microscopic and macroscopic tumor-free resection. The secondary endpoints included postoperative pathologic stage, complete response (CR; no evidence of residual disease) rate, and safety. The postoperative pathologic stage was assessed according to the 7th edition of Union International Contre le Cancer (UICC)/TNM staging system (TNM7) (24). Tumor response was assessed based on the Response Evaluation Criteria in Solid Tumors (RECIST) version 1.1 (25). Safety was assessed by AEs and postoperative complications. AEs were graded referring to Common Terminology Criteria version 5.0 (CTCAE 5.0).

### Statistical analysis

The sample size calculation was based on the primary endpoint of the R0 surgical resection rate. In this trial, we assumed that the R0 surgical resection rates were 60.0% in the nCRT group and 80.0% in the Anlotinib group. Accordingly, a total of 276 patients (138 per group) would be sufficient with a 2-sided type I error of 5% and a power of 90%, as well as a 10% dropout rate. However, due to the preliminary results noted in this open-label study, and the fact that the patient population included had few treatment options, we decided to publish the data before the completion of the trial.

Efficacy analysis was performed according to the per-protocol set (PPS), which was defined as all patients who completed the neoadjuvant treatment and MIE without serious violation of the protocol. Safety was evaluated based on a safety analysis set (SAS), which defined all patients who completed the MIE, and at least one assessment of safety data. Categorical variables (expressed as numbers and percentages) between the two groups were compared using the χ2 test, while continuous variables (expressed as mean with standard deviation) were compared using Student’s t-test. The SPSS (version 23.0; SPSS Institute. IL., USA) software was used for statistical analysis. The significance level is set at *p*<0.05.

## Results

### Patient characteristics

**Figure 2:**
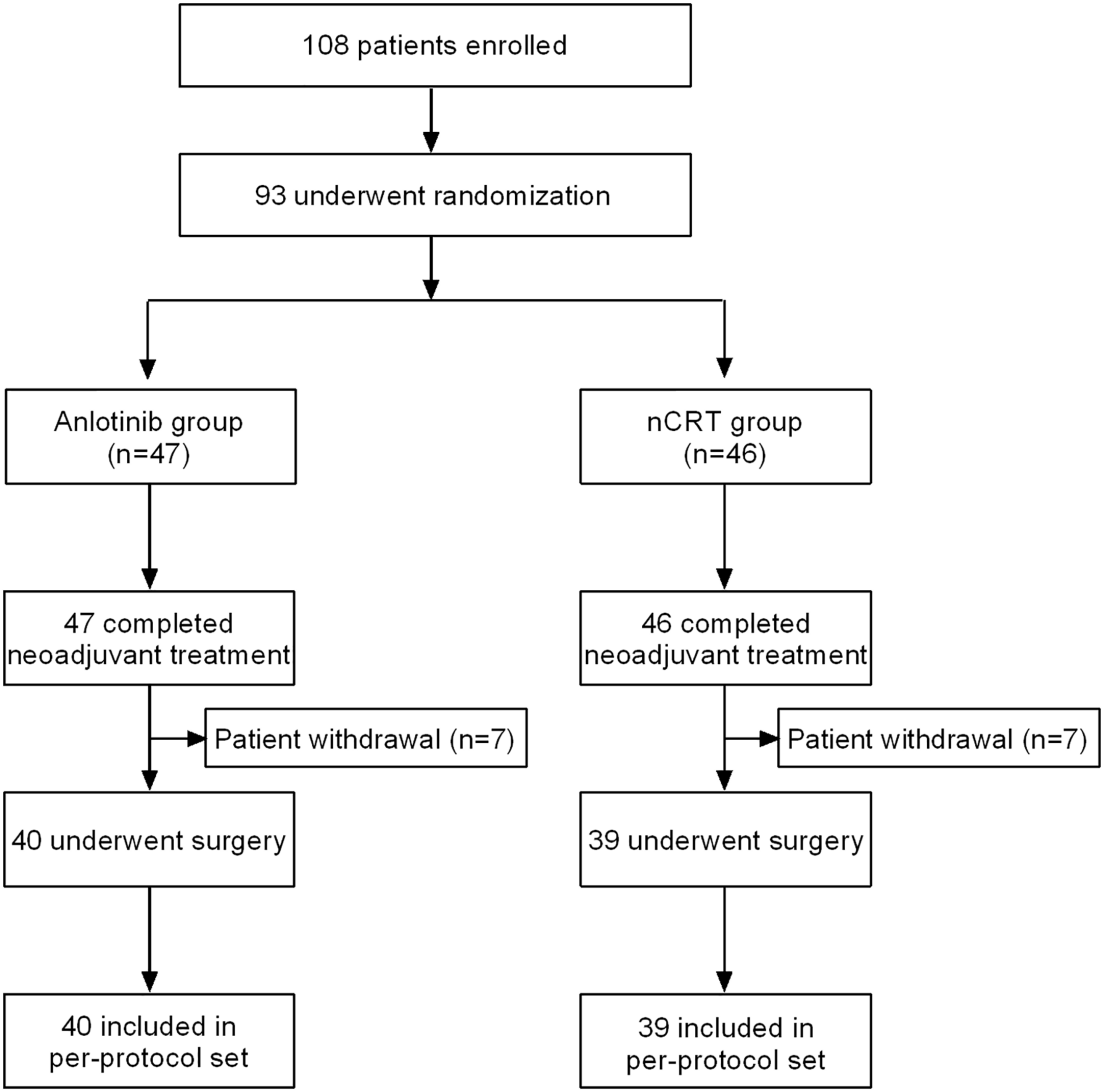
Patients flowchart. Anlotinib group, neoadjuvant Anlotinib combined with chemotherapy; nCRT group, neoadjuvant chemoradiotherapy.

From August 2019 to August 2021, a total of 108 patients (**Figure 2**) were assessed for eligibility, and 93 were eligible and randomly allocated to the nCRT group (n=46) or the Anlotinib group (n=47). Of the 93 patients, all completed neoadjuvant treatment, and 79 underwent MIE and were finally included in the PPS analysis (nCRT group, n=39; Anlotinib group, n=40). The reason for not including in the PPS analysis was patient withdrawal (n=14). Baseline characteristics (**Table 1**) including age, gender, body mass index, smoking history, alcohol abuse history, ECOG performance status, tumor location, and clinical T and N stage were generally well balanced between the two groups (all *p*>0.05).

**Table 1:** Baseline characteristics of the two groups.

Characteristic	Anlotinib group (n = 40)	nCRT group (n = 39)	*p* value
Age (years), median (range)	65.0 (45.0-77.0)	63.0 (47.0-81.0)	0.783
Gender (n, %)			>0.999
Male	35 (87.5)	34 (87.2)	
Female	5 (12.5)	5 (12.8)	
BMI (kg/m^2^, mean ± SD)	22.6 ± 2.9	22.2 ± 2.9	0.971
Smoking history (n, %)	33 (82.5)	33 (84.6)	>0.999
Alcohol abuse history (n, %)	25 (62.5)	18 (46.2)	0.147
ECOG performance status (n, %)			
0	32 (80.0)	34 (87.2)	0.393
1	8 (20.0)	5 (12.8)	
Tumor location (n, %)			0.092
Upper	6 (15.0)	14 (35.9)	
Middle	17 (42.5)	14 (35.9)	
Lower	17 (42.5)	11 (28.2)	
Clinical T stage (n, %)			0.241
cT2	3 (7.5)	0 (0.0)	
cT3	37 (92.5)	39 (100.0)	
Clinical N stage (n, %)			0.843
cN0	15 (37.5)	12 (30.8)	
cN1	16 (40)	17 (43.6)	
cN2	5 (12.5)	7 (17.9)	
cN3	4 (10)	3 (7.7)	

BMI, body mass index; n, number; SD, standard deviations; ECOG, Eastern Cooperative Oncology Group; Anlotinib group, neoadjuvant Anlotinib combined with chemotherapy; nCRT group, neoadjuvant chemoradiotherapy.

### Efficacy

MIE was performed for 39 patients in the nCRT group and 40 patients in the Anlotinib group. Among them, 38 (97.4%) patients in the nCRT group and 40 (100.0%) patients in the Anlotinib group underwent R0 surgical resection (*p*>0.05). The total operation duration (200.7 ± 25.5 vs. 262.2 ± 39.0 min, *p*=0.010) and estimated blood loss (52.4 ± 39.3 vs. 161.3 ± 126.7 mL, *p*<0.001) in the Anlotinib group were significantly lower than that in the nCRT group. There were no significant differences between the two groups in terms of lymph node dissection, retrieved lymph nodes, transfusion, and conversion to open surgery (all *p*>0.05, **Table 2**).

**Table 2:** Surgical outcomes of the two groups.

Factor	Anlotinib group (n = 40)	nCRT group (n = 39)	*p* value
Resection (n, %)			0.494
R0	40 (100.0)	38 (97.4)	
R1	0 (0.0)	1 (2.6)	
Lymph node dissection (n, %)			0.378
Three-field	5 (12.5)	8 (20.5)	
Two-field	35 (87.5)	31 (79.5)	
Retrieved lymph nodes (n, %)			
Total	31.9 ± 11.5	30.6 ± 12.3	0.653
Station	11.3 ± 1.6	10.9 ± 2.8	0.465
Positive	1.5 ± 2.2	1.4 ± 2.0	0.767
The ratio between positive and total lymph nodes	0.06 ± 0.09	0.04 ± 0.07	0.473
Transfusion (n, %)			0.494
Yes	0 (0.0)	1 (2.6)	
No	40 (100.0)	38 (97.4)	
Operation duration (min, mean ± SD)			
Total	200.7 ± 25.5	262.2 ± 39.0	0.010
Thoracoscopic duration	66.4 ± 16.8	106.0 ± 27.9	0.002
Laparoscopic duration	39.6 ± 10.0	39 ± 11.6	0.356
Estimated blood loss (mL, mean ± SD)	52.4 ± 39.3	161.3 ± 126.7	<0.001
Conversion to open surgery (n, %)	0 (0.0)	1 (2.6)	0.494

SD, standard deviations; n, number; Anlotinib group, neoadjuvant Anlotinib combined with chemotherapy; nCRT group, neoadjuvant chemoradiotherapy.

Three of 39 patients (7.7%) in the nCRT group and 4 of 40 patients (10.0%) in the Anlotinib group achieved a pCR, with no statistically significant difference between the two groups (*p*>0.999). As shown in **Table 3**, the pathologic outcomes were similar between the two groups (all *p*>0.05).

**Table 3:** Pathologic outcomes of the two groups.

Variables	Anlotinib group (n = 40)	nCRT group (n = 39)	*p* value
Pathological T stage (n, %)			
ypT0	4 (10.0)	4 (10.3)	>0.999
ypTis	1 (2.5)	3 (7.7)	0.359
ypT1	12 (30.0)	5 (12.8)	0.099
ypT2	9 (22.5)	13 (33.3)	0.323
ypT3	14 (35.0)	14 (35.9)	>0.999
Pathological N stage (n, %)			
ypN0	19 (47.5)	21 (53.8)	0.655
ypN1	10 (25.0)	8 (20.5)	0.790
ypN2	7 (17.5)	9 (23.1)	0.586
ypN3	4 (10.0)	1 (2.6)	0.359
pCR (n, %)	4 (10.0)	3 (7.7)	>0.999
Differentiation (n, %)			
Gx	5 (12.5)	6 (15.4)	0.756
G1	3 (7.5)	3 (7.7)	>0.999
G2	25 (62.5)	17 (43.6)	0.117
G3	7 (17.5)	13 (33.3)	0.126
TRG (n, %)			
0	5 (12.5)	4 (10.3)	>0.999
1	9 (22.5)	7 (17.9)	0.781
2	12 (30)	9 (23.1)	0.612
3	14 (35)	19 (48.7)	0.258
Lymphovascular invasion (n, %)	11 (27.5)	13 (33.3)	0.575
Perineural invasion (n, %)	9 (22.5)	12 (30.8)	0.409

Anlotinib group, neoadjuvant Anlotinib combined with chemotherapy; nCRT group, neoadjuvant chemoradiotherapy; pCR, pathological complete response (ypT0N0M0); TRG, tumor regression grade (TRG 0, pathologic complete response in the primary tumor, showed absence of residual cancer and fibrosis extending through the different layers of the esophageal wall; TRG 1, the presence of rare residual cancer cells scattered through the fibrosis; TRG 2, an increase in the number of residual cancer cells, but fibrosis still predominated; TRG 3, residual cancer outgrowing fibrosis).

Seven of 39 patients (17.9%) in the nCRT group and 5 of 40 patients (12.5%) in the Anlotinib group achieved a CR (*p*>0.05). Besides, no significant differences were found in rates of partial response (61.5% vs. 55.0%), stable disease (17.9% vs. 25.0%), and progressive disease (2.6% vs. 7.5%) between the two groups (all *p*>0.05, **Table 4**). The ORR was similar between the two groups (67.5% vs. 79.4%, *p*>0.05).

**Table 4:** Tumor response of the two groups.

Variables	Anlotinib group (n = 40)	nCRT group (n = 39)	*p* value
Tumor response (n, %)			
Complete response	5 (12.5)	7 (17.9)	0.546
Partial response	22 (55.0)	24 (61.5)	0.650
Stable disease	10 (25.0)	7 (17.9)	0.586
Progressive disease	3 (7.5)	1 (2.6)	0.615
ORR	27 (67.5)	31 (79.4)	0.301

Anlotinib group, neoadjuvant Anlotinib combined with chemotherapy; nCRT group, neoadjuvant chemoradiotherapy; ORR, objective response rate.

### Safety

Treatment-related hematologic and non-hematologic toxicity observed in the two groups is listed in **Table 5**. Overall, treatment-related AEs of any grade were reported in 36 (92.3%) patients in the nCRT group, and 34 (80.0%) patients in the Anlotinib group (*p*=0.481). The majority of AEs were grade 1-2 in the two groups. The frequency and severity of AEs occurred in the two groups were similar (all *p*>0.05).

**Table 5:** Treatment-related adverse events.

Adverse events	Anlotinib group (n = 40)				nCRT group (n = 39)			*p* value
	Grade 1-2	Grade 3	Grade 4		Grade 1-2	Grade 3	Grade 4	
Total (n, %)	27 (67.5)	12 (30.0)	3 (7.5)		29 (74.4)	13 (33.3)	4 (10.3)	0.964
Hematological toxicity (n, %)								
Anemia	17 (42.5)	2 (5.0)	1 (2.5)		17 (43.6)	1 (2.6)	1 (2.6)	0.857
Leukopenia	20 (50.0)	2 (5.0)	0 (0.0)		19 (51.2)	1 (2.6)	0 (0.0)	0.146
Lymphopenia	4 (10.0)	1 (2.5)	0 (0.0)		3 (8.1)	1 (2.6)	0 (0.0)	0.866
Neutropenia	7 (17.5)	2 (5.0)	0 (0.0)		7 (17.9)	2 (5.1)	1 (2.6)	0.622
Thrombocytopenia	12 (30.0)	3 (7.5)	1 (2.5)		13 (30)	2 (5.1)	1 (2.6)	0.887
Non-hematological toxicity (n, %)								
Anorexia	24 (60.0)	10 (25.0)	1 (2.5)		28 (71.8)	10 (25.6)	1 (2.6)	0.955
Alopecia	6 (15.0)	1 (2.5)	0 (0.0)		5 (12.8)	3 (7.7)	0 (0.0)	0.327
Constipation	5 (12.5)	1 (2.5)	0 (0.0)		4 (10.3)	1 (2.6)	0 (0.0)	0.892
Diarrhea	4 (10.0)	1 (2.5)	0 (0.0)		4 (10.3)	1 (2.6)	0 (0.0)	>0.999
Fatigue	10 (25.0)	3 (7.5)	0 (0.0)		12 (30.8)	6 (15.4)	0 (0.0)	0.541
Hepatic dysfunction	4 (10.0)	1 (2.5)	1 (2.5)		3 (7.7)	1 (2.6)	1 (2.6)	0.974
Nausea	27 (67.5)	3 (7.5)	0 (0.0)		29 (74.4)	4 (10.3)	0 (0.0)	0.791
Vomiting	20 (50.0)	12 (30.0)	1 (2.5)		19 (48.7)	11 (28.2)	1 (2.6)	0.997

Anlotinib group, neoadjuvant Anlotinib combined with chemotherapy; nCRT group, neoadjuvant chemoradiotherapy.

The total postoperative complication rates were 30.8% (12/39) in the nCRT group and 22.5% (9/40) in the Anlotinib group (*p*=0.453). The most frequently observed postoperative complications were pleural effusion (20.5% vs. 12.5%), arrhythmia (17.9% vs.10.0%), anastomotic leakage (12.8% vs. 7.5%), recurrent nerve palsy (12.8% vs. 7.5%), and venous thrombosis (10.3% vs. 5.0%) in the nCRT group and Anlotinib group (all *p*>0.05, **Table 6**). The rates of re-operation were 2.6% (1/39) in the nCRT group, and 0.0% (0/40) in the Anlotinib group (*p*>0.05). The reason for re-operation in the nCRT group was the treatment of hemorrhage from an anastomotic fistula in the neck. In-hospital mortality was not observed in any group.

**Table 6:** Postoperative complications.

Variables	Anlotinib group (n=40)	nCRT group (n=39)	*p* value
Postoperative complication (n, %)			
Pleural effusion	5 (12.5)	8 (20.5)	0.378
Arrhythmia	4 (10.0)	7 (17.9)	0.348
Anastomotic leakage	4 (7.5)	5 (12.8)	0.737
Recurrent nerve palsy	3 (7.5)	5 (12.8)	0.481
Venous thrombosis	2 (5.0)	4 (10.3)	0.432
Mediastinal abscess	2 (5.0)	3 (7.7)	0.675
Pneumothorax	1 (2.5)	3 (7.7)	0.359
Pneumonia	1 (2.5)	2 (5.1)	0.615
Wound infection	1 (2.5)	1 (2.6)	>0.999
Atelectasis	0 (0.0)	2 (5.1)	0.241
Bowel obstruction	1 (2.5)	0 (0.0)	>0.999
Re-operation (n, %)	0 (0.0)	1 (2.6)	0.494
In-hospital mortality (n, %)	0 (0.0)	0 (0.0)	>0.999

Anlotinib group, neoadjuvant Anlotinib combined with chemotherapy; nCRT group, neoadjuvant chemoradiotherapy.

## Discussion

To our knowledge, this work was the first available well-designed randomized, phase II trial evaluating the efficacy and safety of neoadjuvant Anlotinib plus nCT followed by MIE for the treatment of patients with locally advanced ESCC. The initial result demonstrated that patients who received preoperative neoadjuvant Anlotinib plus nCT had a similar safety profile and pathologic response, but shorter total operation duration and less blood loss, compared with those who underwent nCRT alone. Thus, the novel neoadjuvant regime of Anlotinib plus nCT followed by MIE seems to be feasible, safe, and effective for the treatment of patients with locally advanced ESCC.

In the last decades, accumulating evidence had illustrated that malignant esophageal tumors can obtain survival benefits from neoadjuvant therapy with chemoradiotherapy or chemotherapy (4–7, 26). However, although patients with locally advanced ESCC have a good pathologic response to nCRT, it also increases the technical difficulty of operation. The formation of fibrosis after nCRT makes it difficult to enter into the appropriate surgical plane, and the arteries are not fade out easily especially when the primary tumor is located in the upper third of the esophagus (27, 28). For patients with poor physical condition, radiation therapy may induce severe injury to the esophagus and adjacent organs, resulting in severe complications such as anastomotic leakage, pneumonia, and cardiotoxicity (29, 30). Besides, nCT cannot achieve a satisfactory pathologic response in patients with esophageal cancer, and even leads to the disease progression in patients with low sensitivity to chemotherapy (9, 10). Hence, selecting the optimal neoadjuvant regimen based on the above contradictory results is challenging. In general, an optimal neoadjuvant regimen should be less technically demanding, safer, more effective, and able to operate smoothly in any institution. Based on this consideration, a novel neoadjuvant regime of Anlotinib plus nCT was implemented in the present study to evaluate the efficacy and safety of this neoadjuvant regimen followed by MIE for the treatment of patients with locally advanced ESCC. Our study was expected to find a novel neoadjuvant regime for patients with locally advanced ESCC by comparing it with nCRT alone, which could not only ensure good pathologic response and acceptable safety, but also reduce the technical difficulty of MIE.

As a novel multitarget TKI, the clinical benefit of Anlotinib has been proved in a variety of tumors, including non-small-cell lung cancer (12), soft tissue sarcoma (13), hepatocellular carcinoma (14), and medullary thyroid cancer (15). In the present study, we evaluated the efficacy of neoadjuvant Anlotinib combined with nCT for patients with locally advanced ESCC by comparing it with nCRT alone. Our results showed that the R0 resection rate (100% vs. 97.4%), pathologic outcomes, and CR rate (12.5% vs. 17.9%) were similar between the two groups. The R0 resection rate observed in patients who received neoadjuvant Anlotinib plus nCT was also consistent with that previously observed in nCT (92.9%-96.2%) (20, 31) and nCRT (93.4%-98.4%) (20, 31, 32). The pCR rates were 7.7% in the nCRT group, and 10.0% in the Anlotinib group, which were lower than that in the CROSS (29% in the nCRT group) and NEOCRTEC5010 (43.2% in the nCRT group) study. It has been well established that the number of lymph node metastases is the strongest prognostic parameter in esophageal cancer (33, 34). The high proportion of patients with the T3N1-2 stage in our study might explain the low pCR rates. It was worth noting that patients who received neoadjuvant Anlotinib plus nCT in our study had less blood loss (52.4 ± 39.3 vs. 161.3 ± 126.7 mL) compared with those who underwent nCRT alone, which may be attributed to the anti-tumor angiogenesis effect of Anlotinib (35). Moreover, the total operation duration (200.7 ± 25.5 vs. 262.2 ± 39.0 min) in the Anlotinib group was shorter than that in the nCRT group. The absence of radiotherapy-induced fibrosis, as well as the reduction of blood loss, made patients who received neoadjuvant Anlotinib plus nCT had a better surgical visualization, which finally resulted in a shorter total operation duration compared with nCRT alone. Taken together, in addition to antitumor activity, the neoadjuvant regimen containing Anlotinib and chemotherapy had the additional benefits of reducing the technical requirement of the MIE procedure compared to nCRT alone in patients with locally advanced ESCC.

In the present study, the neoadjuvant regimen of Anlotinib plus nCT was well tolerated for patients with locally advanced ESCC. The AEs observed in the Anlotinib group were generally consistent with those described in previous reports with nCT alone (7, 36), indicating that this neoadjuvant regimen did not cause an additional risk of toxicity. Compared with those who received nCRT plus surgery, patients who were treated with Anlotinib combined with nCT followed by surgery had a similar incidence of AEs (92.3% vs. 80.0%) and postoperative complications rate (30.8% vs. 22.5%). In addition, the majority of AEs in the two groups were regarded as grade 1-2 (74.4% vs. 67.5%). Although hemorrhage/bleeding (37) and hypertension (38) were the most common AEs of anlotinib, no patients in the present study experienced these AEs. Patients with a history of hypertension taking antihypertensive drugs regularly might attribute to the low incidence of hypertension. It was worth noting that both the postoperative complications rate (22.5% vs. 42.6) and in-hospital mortality rate (0.0% vs. 3.8%) observed in the Anlotinib group were slightly lower than that occurred in the previous reports (9, 20). This may be attributed to the fact that centers in China have developed more extensive clinical experience in the treatment of ESCC, due to the high incidence and prevalence of ESCC in China, and operations were usually performed in the large-capacity medical center. Overall, the safety profile of neoadjuvant Anlotinib plus nCT followed by MIE was acceptable and well-tolerated in patients with locally advanced ESCC.

There were several limitations in the current study. Firstly, due to the preliminary results noted in this open-label study, and the fact that the patient population included had few treatment options, the study closed early for slow accrual. Secondly, the long-term survival benefits such as DFS and OS were not completely mature due to the short follow-up period, and the final results will be presented when the data fully mature. Thirdly, patients in our study were only recruited from a single center in China, which might limit the generalization of our results to other geographic regions or racial backgrounds. Therefore, further study in wider populations is warranted to validate the efficacy of neoadjuvant Anlotinib plus nCT followed by MIE for the treatment of patients with locally advanced ESCC.

## Conclusion

The first published randomized controlled trial demonstrated that the neoadjuvant regimen containing Anlotinib and chemotherapy showed favorable surgical outcomes and a similar safety profile and pathologic response for patients with locally advanced ESCC compared with nCRT alone, with shorter total operation duration and less blood loss. These findings suggest that preoperative neoadjuvant Anlotinib plus nCT might reduce the technical requirement of the MIE procedure, and might be a potential neoadjuvant therapy option for locally advanced ESCC.

## Data availability statement

The original contributions presented in the study are included in the article/supplementary material. Further inquiries can be directed to the corresponding authors.

## Ethics statement

This study was conducted in conformance with Good Clinical Practice guidelines and the Declaration of Helsinki, and approved by the ethics committee of the Daping Hospital, Army Medical University (Third Military Medical University) (number: 202044). The patients/participants provided their written informed consent to participate in this study. Written informed consent was obtained from the individual(s) for the publication of any potentially identifiable images or data included in this article.

## Author contributions

Conception and design, WG and Z-YZ; Provision of study materials or samples, Y-JW, K-KL, X-FX, TB, Z-PH, JL, and SW; Collection and assembly of data, Y-JW; Data analysis and interpretation, Y-JW; Drafting article, Y-JW; Administrative support, WG. All the authors have read and approved the final manuscript.

## Conflict of interest

The authors declare that the research was conducted in the absence of any commercial or financial relationships that could be construed as a potential conflict of interest.

## Publisher’s note

All claims expressed in this article are solely those of the authors and do not necessarily represent those of their affiliated organizations, or those of the publisher, the editors and the reviewers. Any product that may be evaluated in this article, or claim that may be made by its manufacturer, is not guaranteed or endorsed by the publisher.
